# Compensatory contribution of retinal larger vessels to perfusion density in diabetics without retinopathy

**DOI:** 10.1038/s41598-021-02554-y

**Published:** 2022-01-10

**Authors:** Dulce Milagros Razo Blanco-Hernández, Selma Alin Somilleda-Ventura, Rebeca Chávez-Herrera, María Guadalupe Colas-Calvere, Virgilio Lima-Gómez

**Affiliations:** 1 Research Division, Hospital Juarez de Mexico, 07760 Mexico City, Mexico; 2 Biomedical Research Center, Fundacion Hospital Nuestra Señora de La Luz IAP, 06030 Mexico City, Mexico; 3 Medicine School, Universidad Autonoma de Ciudad Juarez, 32315 Ciudad Juarez, Mexico; 4 Ophthalmology Service, Hospital Juarez de Mexico, 07760 Mexico City, Mexico

**Keywords:** Endocrine system and metabolic diseases, Diseases, Endocrinology, Signs and symptoms

## Abstract

Vessel and perfusion densities may decrease before diabetic retinopathy appears; it is unknown whether these changes affect the contribution of vessel density to perfusion density. This was a non-experimental, comparative, prospective, cross-sectional study in non-diabetic subjects (group 1) and diabetics without retinopathy (group 2). Vessel and perfusion densities in the superficial capillary plexus were compared between groups at the center, inner, and full regions and by field (superior, temporal, inferior, nasal) using optical coherence tomography angiography. Coefficients of determination (R^2^) between vessel and perfusion densities were calculated to find the contribution of larger retinal vessels to perfusion density. Percent differences were used to evaluate the contribution of these vessels to perfusion density in a regression model. There were 62 participants, 31 eyes by group; vessel and perfusion densities as well as the coefficients of determination between them were lower in group 2, especially in the nasal field (R^2^ 0.85 vs. 0.71), which showed a higher contribution of larger retinal vessels to perfusion density. The regression model adjusted to a quadratic equation. In diabetics without retinopathy the contribution of vessel density to perfusion density may decrease; a low vessel density may increase the contribution of larger retinal vessels to perfusion density.

## Introduction

Optical coherence tomography angiography (OCTA) allows to visualize retinal vessels and their flow without requiring intravenous contrast, which makes it possible to assess the capillary plexuses with measurements such as perfusion density, vessel density, foveal avascular zone area and intercapillary spaces, among others^1,2^. The Cirrus Angioplex OCTA device (Zeiss, Dublin CA) measures perfusion density as the percentage area where there is circulation, and vessel density as the length of vessels with circulation in a defined area^3,4^; according to Fawzi, vessel density removes the effect of vessel diameter and any extra effect carried by larger retinal vessels in the superficial network^5^.

A study in healthy people from our population found a coefficient of determination of R^2^ = 0.98 between center vessel density and center perfusion density^6^; only 2% of the changes of perfusion density in the central macula could be explained by large vessel diameter or dilatation, because this region has few vessels that are not capillaries. Vessel density is a biomarker of capillary status, that retinal vascular diseases like diabetes can decrease; the changes of larger vessel diameter and dilatation that happen as vessel density decreases could be estimated by a coefficient of determination between vessel and perfusion densities.

Although it has already been reported that vessel and perfusion densities may decrease in diabetes before diabetic retinopathy (DR) appears^7–9^, and one study found that perfusion density increased^10^, there is few information about the proportion in which vessel density contributes to perfusion density, which could indirectly allow to know whether larger retinal vessels change when capillary damage begins. We conducted a study to compare the contribution of vessel density to perfusion density between subjects without diabetes and diabetic patients without DR, using OCTA.

## Results

The sample consisted of 62 eyes from 62 patients, 31 in each group. Group 1 had a median age of 45 years (interquartile range [IQR] 34–60), 16 subjects were female (51.6%), and 17 eyes were right (54.8%). In group 2 median age was 55 years (IQR 49–57), 19 subjects were female (61.3%), and 12 eyes were left (38.7%). Table 1 presents the comparison between groups.

**Table 1:** Demographic variables by group.

	Group 1 (n = 31)	Group 2 (n = 31)	*p*
Age (years)	45 (34–60)	55 (49–57)	0.063*
Female gender	16 (51.6%)	19 (61.3%)	0.44**
Diabetes duration (years)	–	7.65 ± 5.84	–
HbA1c (%)	–	7.9 ± 2.31	–
Spherical equivalent (diopters)	+ 1.00 (0.00– + 3.00)	+ 1.75 (0.00– + 2.50)	0.87*

*Mann–Whitney’s U.

**χ^2^.

**Figure 1:**
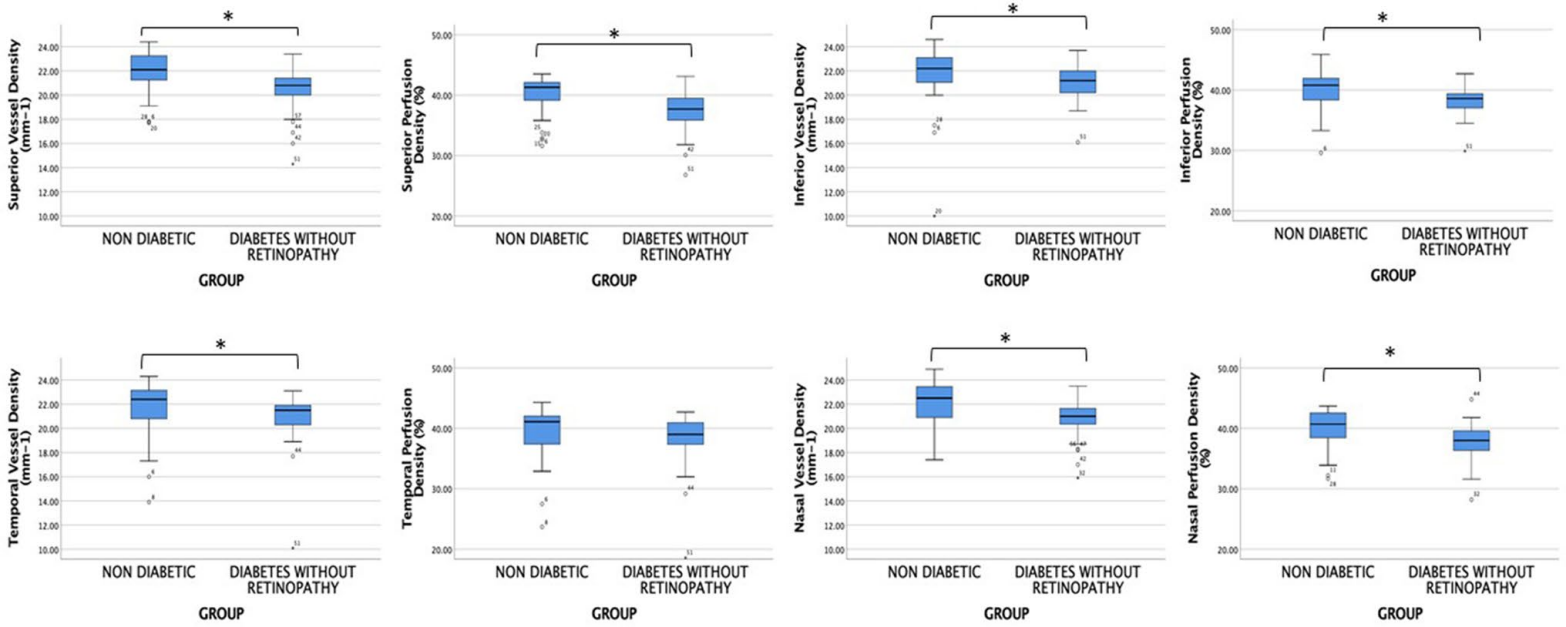
Vessel and perfusion densities by field in each group. Boxes stand for the interquartile range of values in each group. *p < 0.05.

Vessel and perfusion densities in each region and field were statistically lower in group 2 than in group 1 (Fig. 1), except by perfusion density in the temporal field (Table 2). The values ​​of the foveal avascular zone did not differ between the groups; the comparison of vessel and perfusion densities by field within each group did not show statistical differences (*p* < 0.05).

**Table 2:** Variable distribution by group.

Variable	Group 1 (n = 31) Median (25th and 75th percentile)	Group 2 (n = 31) Median (25th and 75th percentile)	p*
Center vessel density (mm^−1^)	11.6 (8.3–12.7)	8.8 (7.5–11.6)	0.046
Inner vessel density (mm^−1^)	22.5 (21.5–23.0)	20.8 (20.2–21.7)	0.002
Full vessel density (mm^−1^)	21.2 (19.9–21.7)	19.6 (18.9–20.3)	0.002
Superior vessel density (mm^−1^)	22.1 (21.1–23.3)	20.8 (19.9–21.5)	0.001
Temporal vessel density (mm^−1^)	22.4 (20.8–23.2)	21.5 (20.1–22.0)	0.009
Inferior vessel density (mm^−1^)	22.2 (21–23.1)	21.2 (20.2–22.2)	0.023
Nasal vessel density (mm^−1^)	22.5 (20.9–23.5)	21.0 (20.3–21.7)	0.004
FAZ** area (mm^2^)	0.25 (0.21–0.37)	0.29 (0.20–0.38)	0.827
FAZ** perimeter (mm)	2.25 (1.99–2.58)	2.3 (2.05–2.65)	0.657
FAZ** circularity	0.68 (0.62–0.73)	0.67 (0.56–0.72)	0.617
Center perfusion density (%)	20.0 (14.8–22.5)	14.9 (13.0–20.2)	0.034
Inner perfusion density (%)	40.7 (37.8–41.7)	38.2 (36.9–39.3)	0.003
Full perfusion density (%)	38.4 (36.4–39.4)	35.4 (34.5–37.0)	0.001
Superior perfusion density (%)	41.3 (38.1–42.2)	37.7 (35.8–39.5)	0.009
Temporal perfusion density (%)	41.1 (37.3–42.1)	39.0 (37.2–41.0)	0.059
Inferior perfusion density (%)	40.8 (38.2–42)	38.6 (36.5–39.5)	0.006
Nasal perfusion density (%)	40.7 (37.6–42.7)	38.0 (36.2–39.7)	0.003

*Mann–Whitney’s U.

**Foveal avascular zone.

**Figure 2:**
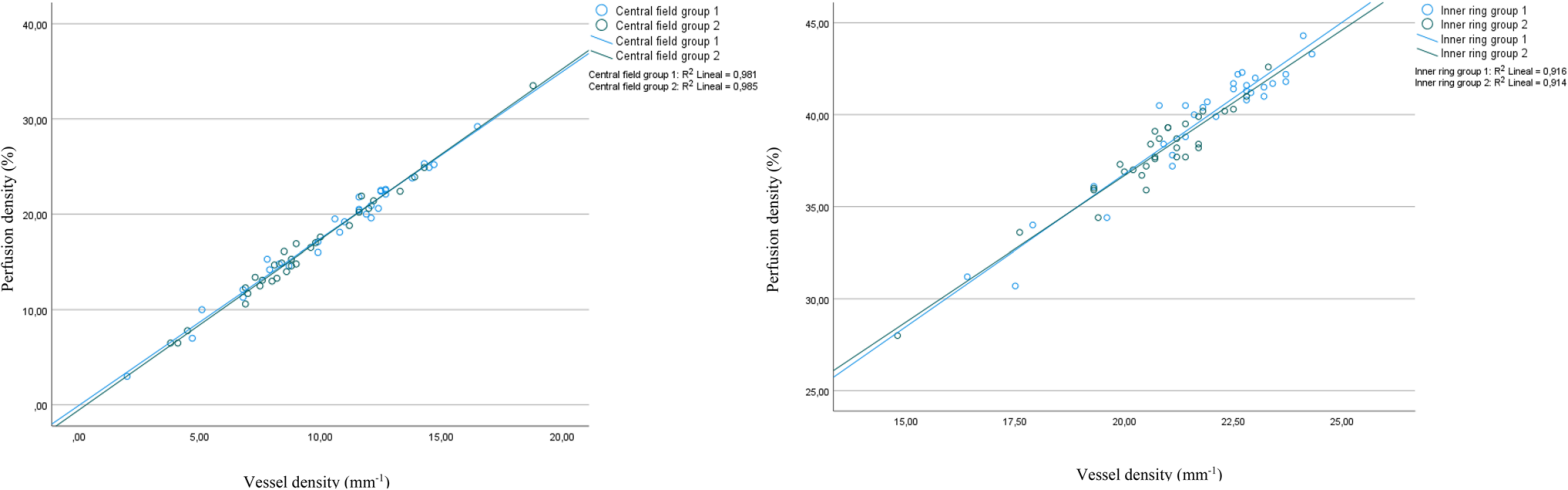
Scatterplot and regression lines between center and inner vessel and perfusion densities in each group. Center densities had no difference because this region has few larger retinal vessels; inner densities showed no difference in R^2^ either, as their values are the sum of the four fields.

There were strong correlations between vessel and perfusion densities in both groups; the comparison of these correlations between fields within each group did not find statistical differences. The coefficients of determination between both variables for each region and field are presented in Table 3; Fig. 2 shows the comparison of regression lines between center and inner vessel and perfusion densities, which did not differ between groups.

**Table 3:** Correlations and coefficients of determination between variables in each group.

Vessel density/perfusion density	Group 1		Group 2	
	Spearman’s Rho	Coefficient of determination (R^2^)	Spearman’s Rho	Coefficient of determination (R^2^)
Center	0.98*	0.98	0.97*	0.98
Inner	0.89*	0.91	0.89*	0.91
Full	0.93*	0.94	0.93*	0.93
Superior	0.83*	0.82	0.87*	0.92
Temporal	0.82*	0.93	0.84*	0.91
Inferior	0.85*	0.93	0.87*	0.86
Nasal	0.87*	0.85	0.84*	0.71

*p < 0.001.

**Figure 3:**
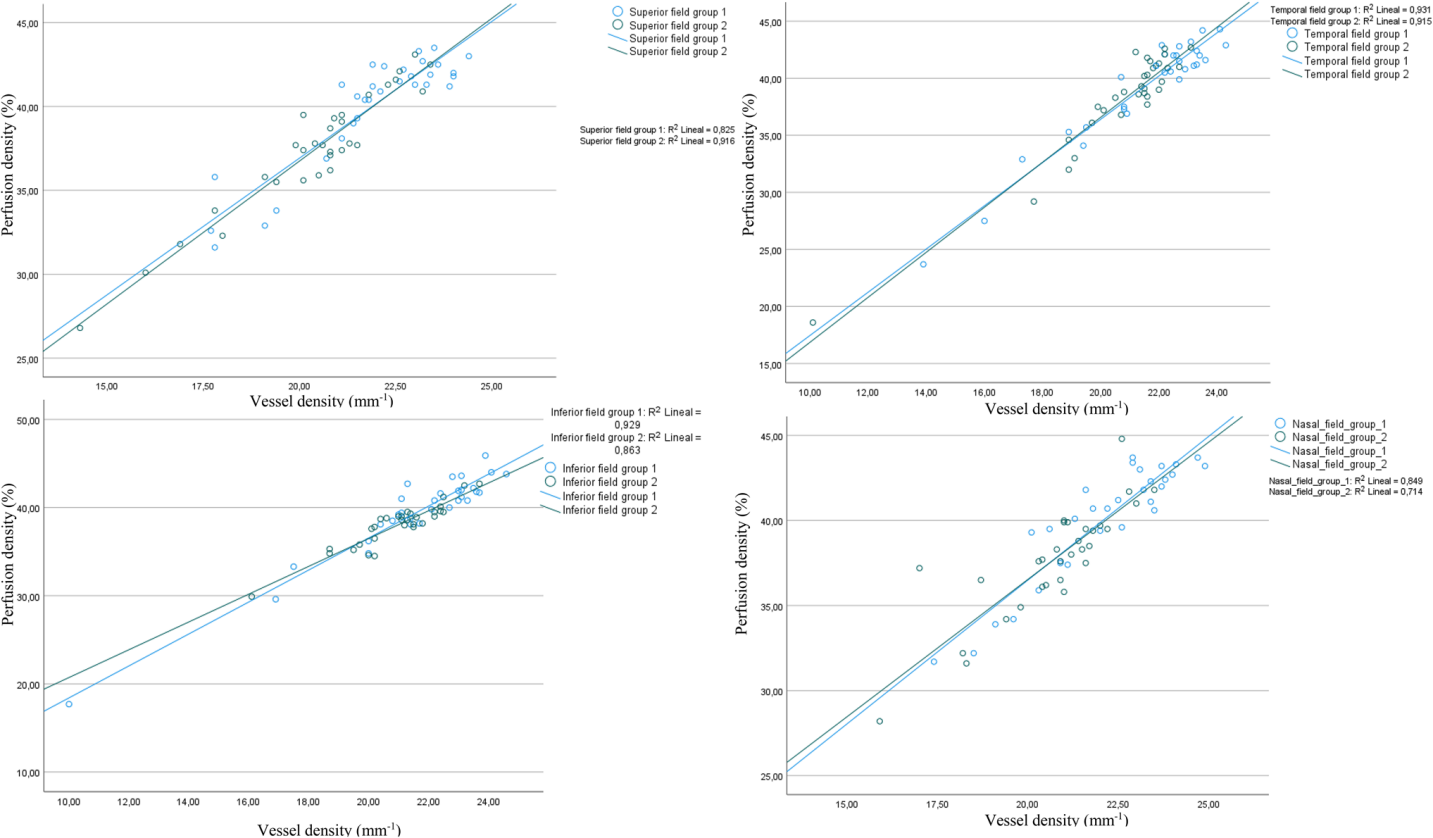
Scatterplot and regression lines between vessel and perfusion densities in each group, by field. There were regional differences that showed a different contribution of larger retinal vessels to perfusion density.

The comparison of coefficients of determination for each field between groups found differences that did not appear when the inner densities were evaluated. Figure 3 shows the comparison of regression lines between vessel and perfusion densities by field: there were lower R^2^ values in the inferior and nasal fields of group 2, and a lower R^2^ value in the superior field of group 1.

**Figure 4:**
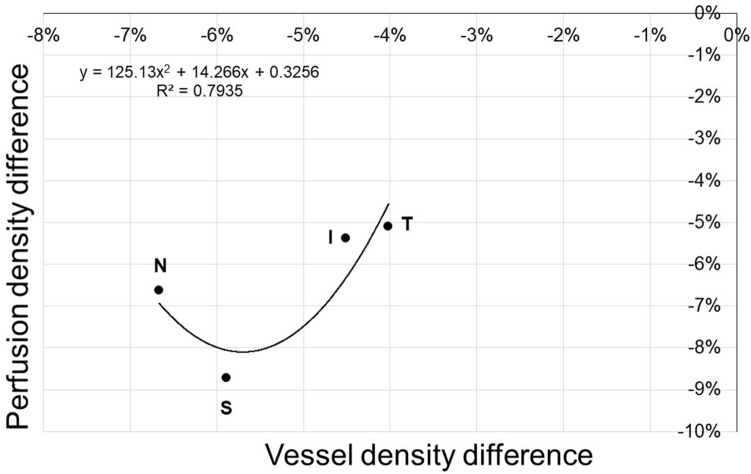
Scatterplot of percent differences of median vessel and perfusion densities values in group 2, with respect to group 1. Although the nasal field had the lowest vessel density its perfusion density was higher than that of the superior field, which had a larger vessel density. The coefficient of determination between vessel and perfusion density was also the lowest in the nasal field (Fig. 3), which showed a higher contribution of retinal larger vessels to its perfusion density. The quadratic model suggests that there could be a vasodilatation after vessel density drops beyond an inflection point, to improve perfusion density. *S* superior field *T* temporal field, *I* inferior field, *N* nasal field.

The percent difference of vessel and perfusion density medians between groups by field is presented in Fig. 4. Though no field had a better correlation between vessel and perfusion densities within each group, the percent differences of the medians of both variables grew simultaneously from the temporal field towards the inferior and the superior fields. The nasal field had a larger percent difference of median vessel density and a shorter percent difference of median perfusion density, which did not adjust to the linear behavior that the other three fields had. The regression analysis showed that the better fit corresponded to a quadratic equation, with a 0.79 coefficient of determination.

## Discussion

Diabetic patients without retinopathy had lower vessel and perfusion densities than healthy subjects, both globally and by field. The differences between groups varied in each field and corresponded mainly to capillary changes.

Finding a lower vessel density in eyes without diabetic retinopathy was consistent with studies that have used the same OCTA device^11–13^. As in the study if Oliverio et al.^14^, foveal avascular zone parameters did not differ between groups.

Vessel density in diabetic patients varied topographically with respect to the reference group: it was progressively lower statistically in the inferior, temporal, nasal, and superior fields. Lee had also found a larger difference of vessel density between non-diabetic subjects and diabetics without retinopathy in the superior field^9^, and Uğurlu reported a lower vessel density in the superior field of diabetics without retinopathy, using another OCTA device^15^; however, this distribution might be random, because there is not a preferential location for diabetic-related capillary damage.

All the medians of vessel density and even the 25th percentile in group 2 had higher values ​​ than the cut-off point proposed by Barraso for inner vessel density in patients with type 1 diabetes (19.15 mm^−1^); however, the 25th percentile values of perfusion density in the superior, inferior, and nasal fields ​​were lower than the cut-off point reported by this author for inner perfusion density (37%)^16^.

The coefficient of determination between vessel and perfusion densities allowed to find the proportion of the area with circulation that changes in length of perfused vessels did not explain; this measurement showed that vessel diameter in two fields had a larger contribution to perfusion density in diabetic patients, than in healthy subjects. A possible explanation for this difference could be vasodilatation; capillaries and venules dilate 1%, arterioles dilate 6% after flicker stimulation^17^, so the increased contribution of vessel dilatation to perfusion density probably resulted of arterial dilatation: however, Singer^18^ and Kushner-Lenhoff^19^ have found that OCTA can measure capillary dilatation under acute hypercapnic conditions.

In the superior and temporal fields of diabetics without retinopathy there was no vasodilation, because the coefficient of determination between vessel and perfusion densities was not lower than in non-diabetic subjects; in the nasal field, the coefficient of determination between vessel and perfusion densities in group 2 was 14 percentage points lower than in group 1, which shows that the area with circulation had a larger contribution of vessel dilatation.

This dilatation could correspond to the increased parafoveal perfusion density that Sousa et al. described in healthy subjects with a stimulus that induces acute hypoxia^20^; however, the temporal and superior fields in diabetic patients did not show vasodilation, despite having lower vessel densities than those of non-diabetic subjects. This was consistent with the lack of vasodilation that Sousa et al. reported in patients with type 1 diabetes, with the same stimulus that induces acute hypoxia^21^, and would suggest opposite responses of vessel diameter to a reduction in vessel density.

When we looked for a rate of change in vessel and perfusion densities, we found that the largest percent difference of the medians between groups was that of vessel density in the nasal field, and of perfusion density in the superior field. Percent difference of medians between groups progressed from temporal to inferior, superior, and nasal fields; that of perfusion density progressed from inferior to nasal and superior fields and was shorter in the nasal field, where there was the largest difference of coefficient of determination between vessel and perfusion densities. This would suggest that, beyond a certain reduction of vessel density, perfusion density would be compensated by arteriole dilatation, as reported by Cheng et al. in induced hypoxia conditions^22^; the percentage difference of the densities by field with respect to the reference group, had a better fit with a parabolic regression line than with a straight one.

Whether the change of vessel perfusion corresponds to vasodilatation needs to be confirmed with other methods that measure arteriolar diameter directly. While that confirmation is available, it would be sensible to use both vessel and perfusion density to evaluate changes of foveal circulation, because their combination offers information that isolated measurements cannot produce; for instance, some studies that used only perfusion density to compare the capillary plexuses between non-diabetics and diabetics without retinopathy could have missed information about reduced vessel density^23–26^, if vasodilatation had compensated the percent of area with circulation.

A strength of the study was the evaluation by fields, which compared regions in eyes with the same systemic characteristics; one more was the automated measurement of vessel and perfusion densities, in studies with signal intensity ≥ 8. The main limitation was the age range, which could change the densities in older subjects; however, the comparison between fields showed differences in the same subject and it was in these measurements where we noticed an increased contribution of larger retinal vessels. We consider that these findings need confirmation in groups with similar age distributions and preferably stratified, to find the contribution of the changes in larger retinal vessels to the changes in perfusion density more accurately.

The lower vessel and perfusion densities and their relationship in patients with diabetes need the evaluation of functional variables in further studies. These findings may be an opportunity for the early identification of events that induce reactive vasodilation, such as hypoxia, with a non-invasive study; they could also help to find characteristics associated with the risk of damage progression, both in retinal capillaries and in other regions of the body.

Calculating the coefficient of determination between vessel and perfusion densities only requires automated measurements from a conventional, commercially available equipment; this is an accessibility advantage with respect to other methodologies and could allow to evaluate more advanced pathological conditions; field analysis allowed to find differences that were undetectable by the conventional evaluation of isolated inner densities.

In conclusion, the coefficient of determination between vessel and perfusion densities changes in diabetics without retinopathy, according to the extent of capillary circulation. A low vessel density with a better perfusion density could be explained by a dilatation of larger retinal vessels.

## Methods

A non-experimental, comparative, prospective, cross-sectional, and open study was conducted in subjects without diabetes and diabetic patients without DR from Mexico City and its metropolitan area. The sample was obtained from patients who attended the ophthalmology service at a federal reference hospital between February 1st and March 31st, 2019. The study adhered to the tenets of the Declaration of Helsinki and received authorization from by the Institutional Review Board (Comité de Investigación del Hospital Juárez de México) of the hospital where it took place (HJM0430 /18-I). All the participants signed a written informed consent.

Subjects aged between 30 and 60 years of any gender, without diabetes or with diabetes who did not have clinical signs of DR and who had an OCTA without significant movement or artifact shadows, with a signal intensity > 7 were included in the study. Subjects with a spherical equivalent >  − 6.00 diopters and other ocular or retinal diseases that could confuse the results (glaucoma, hemorrhages, or cataracts) or alter the quality of the image were not included. All the participants who had structural macular alterations or measurement errors in the OCTA maps were excluded from the study.

All the patients had an ophthalmic evaluation that included: subjective refractive error, visual acuity with the best optical correction measured in Snellen equivalents, and 45° fundus photographs under pharmacological mydriasis (VisuCam lite fundus camera, Zeiss). A single investigator obtained all the OCTA studies under a standardized protocol, and measured vessel and perfusion densities of the superficial capillary plexus of the macula, as well as the characteristics of the foveal avascular zone in a 3 × 3 mm map, with the HD-OCT Cirrus 5000 with AngioPlex (Zeiss, Dublin CA) device, using the OMAG algorithm.

Subjects without diabetes were assigned to group 1, patients with diabetes without DR were assigned to group 2; one eye per patient was selected using a random number program.

Vessel density was defined as the sum of the length of the vessels with circulation found in the foveal 3 × 3 mm map, measured in mm/mm^2^ (mm^−1^); perfusion density was defined as the percentage of the evaluated surface where there was circulation, measured in percentage. Both variables were measured by regions: center (a 1 mm diameter circle concentric to the foveal center), inner (a 1 mm diameter ring, between 0.5 and 1 mm from the foveal center) and full (a 3 mm diameter circle concentric to the foveal center), and by fields in the inner ring: superior, temporal, inferior and nasal. The device automatically generated the measurements of both variables.

As the Kolmogorow-Smirnov test showed that the variables did not have a normal distribution, we worked with medians and interquartile ranges. The values of vessel and perfusion densities​​ were compared between groups using a Mann–Whitney’s U test; the values of both variables were compared between fields within each group using a Kruskall-Wallis test. Subsequently, the correlations between the values of vessel and perfusion densities in every region and field were found for each group, using Spearman’s Rho; a *p* value of < 0.05 was considered a statistical difference; when this value was significant, the coefficient of determination (R^2^) between vessel and perfusion densities was also calculated in each group.

To analyze the rate of change between fields in group 2, the percent difference between the medians of vessel and perfusion densities in each field were calculated, using the value of group 1 as the reference. A regression analysis was used to find the lineal model with the best fit.

Data was stored and analyzed using the IBM SPSS Ver. 26 program (SPSS Inc, Chicago, IL).
